# Effects of *Poria cocos* extract and protein powder mixture on glucolipid metabolism and rhythm changes in obese mice

**DOI:** 10.1002/fsn3.3245

**Published:** 2023-03-09

**Authors:** Qiaoling Xie, Xiuzhen Jia, Wei Zhang, Yuhan Xu, Meizhen Zhu, Zifu Zhao, Jingyu Hao, Haoqiu Li, Jinrui Du, Yan Liu, Haotian Feng, Hongwei Li

**Affiliations:** ^1^ School of Public Health Xiamen University Xiamen China; ^2^ Inner Mongolia Dairy Technology Research Institute Co. Ltd. Hohhot China; ^3^ Yili Innovation Center Inner Mongolia Yili Industrial Group Co., Ltd. Hohhot China

**Keywords:** inflammation, glucolipid metabolism, obesity, *Poria cocos* extract, protein powder mixture, respiratory exchange rate

## Abstract

Herein, we explored the effects of *Poria cocos* extract, protein powder mixture, and their combined intervention on weight loss in high‐fat diet (HFD)‐induced obese mice. Male C57BL/6J mice were selected and fed a HFD for 8 weeks; obese mice that were successfully modeled were divided into modeling and five intervention groups, and given the corresponding treatment for 10 weeks. Body weight, fat, and muscle tissue, blood glucose, lipids, inflammatory factors, and other glucose and lipid metabolism‐related indicators were measured to evaluate the effect of *P*. *cocos* and protein powder intervention on weight loss in obese mice. The body weight of the intervention group was reduced compared with the HFD group. Fat content of mice in F3PM group decreased significantly (*p* < .05). Levels of blood glucose, lipids, adiponectin, leptin, and inflammatory factors, including interleukin‐1 β and tumor necrosis factor‐ α showed improvement. Lipoprotein lipase (lower about 2.97 pg/ml, vs. HFD mice 10.65 mmoL/ml) and sterol regulatory element‐binding transcription factor (lower about 1413.63 pg/ml, vs. HFD mice 3915.33 pg/ml) levels in liver tissue were decreased. The respiratory exchange rate (RER) of mice in the HFD and subject intervention groups had no circadian rhythm and was maintained at approximately 0.80. The protein powder mixture (PM) group had the lowest RER (*p* < .05), the *P*. *cocos* extract (FL) and F1PM groups had similar RER to the HFD group (*p* < .05), and the F2PM group had a higher RER than the HFD group (*p* < .05). And food intake and energy metabolism returned to circadian rhythm, with an increase in the dose of *P*. *cocos* extract, the feeding rhythms of F1PM, F2PM, and F3PM were closer to that of the normal diet (ND) group. Feeding intervention with *P*. *cocos* and protein powder improved fat distribution, glucolipid metabolism, and energy metabolism, with the combination of F3PM showing more diverse benefits.

## Graphical abstract

The intervention of *poria cocos* extract and protein powder can effectively improve the disorder of glucose and lipid metabolism and contribute to the recovery of biological rhythm. Compared with the use of *P*. *cocos* extract and protein powder alone, the combined supplementation of F3PM has more health benefits.

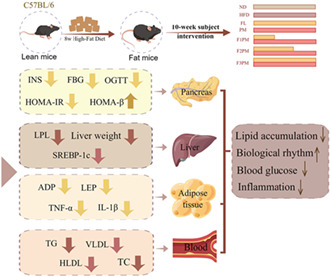

## 1 INTRODUCTION

Obesity is a common metabolic disease that occurs when the body's energy intake exceeds normal physiological needs, causing lipids to form and accumulate in the body (Pan & Myers, 2018). As living standards have modernized, obesity has become the most common chronic metabolic condition in today's society (Purnell, 2000) and it is a public health problem that cannot be ignored. Obesity has become a growing concern since the 1980s, and the rapid increase in obesity rates have placed a heavy burden on the health people around the world (Inoue et al., 2018). Globally, approximately, 1.9 billion adults over 18 are overweight, with over 600 million obese and 42 million children under the age of five being overweight or obese. According to relevant data, it is estimated that by 2025, the global obesity rate will account for one‐fifth of the adult population (Mohammed et al., 2018; World Health Organisation, 2020).

From 2015 to 2019, more than half of China's adult residents were overweight or obese. Additionally, the overweight and obesity rates in children and adolescents over and under six reached 19% and 10.4%, respectively. Presently, we are facing a serious need for obesity prevention and control as the number of obese people increases yearly, the age of onset of obesity advances, and weight control becomes a more arduous task.

Obesity is related to genetics, metabolism, lifestyle, and diet (Wyatt et al., 2006). The condition affects physical appearance and increases the risk of various chronic diseases, such as diabetes, hypertension, coronary heart disease, stroke, and other conditions (Kopelman, 2007; Nikolopoulou & Kadoglou, 2012; Piche et al., 2020); hence, it is one of the biggest threats to human health. In addition, obesity increases the economic burden on individuals and society, and surveys have shown that more than $2 trillion in global healthcare expenditures are due to overweight and obesity (Weerasekara et al., 2016). Furthermore, there is a significant positive correlation between obesity and personal annual medical expenditure, which increases the cost of both direct medical care and the indirect burden, that is, other losses caused by obesity, including the decline in life expectancy, loss of work productivity, reduction in working hours, and reduction in family income (Cecchini & Vuik, 2019).

Traditional Chinese medicine believes obesity is mostly related to spleen deficiency, phlegm, and dampness. *Poria cocos* can permeate dampness and diuresis, invigorate the spleen, and resolve phlegm, thus playing a role in weight loss and fat reduction. *P*. *cocos* is a Chinese herbal medicine that belongs to the dry sclerotium of polyporaceae fungi and is used as medicine and food (Ríos, 2011). The bioactive components of *P*. *cocos* include polysaccharides, triterpenoids, steroids, amino acids, choline, histidine, and potassium salts (Ríos, 2011; Sun, 2014; G. Zhang et al., 2019). Poria polysaccharide, the main component of Poria, accounts for 84% of all components in the dried nucleus (Wang et al., 2011). Studies have shown that *P*. *cocos* extract can inhibit triglyceride (TG) accumulation in cells and activate 5′ adenosine monophosphate‐activated protein kinase to inhibit hepatic steatosis, thus improving fatty liver in obese mice (Kim et al., 2019). In addition, (Zhu et al., 2022) discovered that *P*. *cocos* oligosaccharides improved glucose and lipid metabolism disorders in mice fed a high‐fat diet, and showed improved glucose tolerance and insulin resistance, and inhibition of inflammation. The *P*. *cocos* extract used in this study was from Lipucan®, a patented raw material of XINPHAR GROUP. Related data show that *P*. *cocos* extract can protect muscle cells from injury, promote muscle cell regeneration and promote muscle protein synthesis (Wang et al., 2022). In addition, it also contributes to the absorption of common amino acids in protein, namely, leucine, valine, and isoleucine (branched chain amino acids) (Lin et al., 2009).

Diet restructuring is also important for weight loss and high‐protein dietary interventions have shown to be particularly beneficial. In recent years, mixtures of dairy and soy proteins have been increasingly used in commercial sports nutrition products. Studies have confirmed that high‐protein diets reduce hunger and increase satiety and resting energy expenditure (Moon & Koh, 2020); therefore, the intake of high‐protein diets may reduce voluntary food intake. The ingested protein cannot be stored but is immediately metabolized and utilized in anabolic processes such as peptide synthesis, new protein synthesis, urea production, and gluconeogenesis; hence, the entire metabolic process consumes a large amount of energy. Casein, a whey protein, is one of the two main proteins in milk and has a higher satiety sensation than other protein sources (Westerterp‐Plantenga et al., 2012). Several studies have demonstrated that whey protein suppresses hunger and reduces weight gain and obesity in rats and mice (Pal et al., 2014). Moreover, whey protein supplementation is shown to improve the weight, total fat content, and cardiovascular risk factors in overweight or obese individuals (Pal et al., 2019). As a plant protein, soy protein intake significantly reduces serum cholesterol and TGs concentrations, and compared with whey protein, is rich in glutamine, arginine, and other amino acids (Hursel et al., 2010). In the present study, casein, whey protein, and soy protein isolate were mixed in a ratio of 2:1:1. Studies have shown that this proportion of protein mixture can prolong the synthesis of postprandial skeletal muscle protein (Butteiger et al., 2013). Soy protein has a moderate digestion rate, which can be combined with relatively ‘fast’ digested whey protein and “slow” digested casein to maintain a stable supply of amino acids and promote muscle growth (Paul, 2009). Compared with the simple intake of the same protein, the three proteins are combined so that their unique properties and can exert greater efficacy.

Feeding intervention with *P*. *cocos* and a high‐protein mixture combines medicine and food with the dual effect of pharmacotherapy and food therapy, which can be more advantageous than a single drug intervention. The two approaches can exert anti‐obesity effects by modulating metabolic disorders through multiple interactions in the obesity process rather than simply suppressing appetite. The protein in protein powder and branched chain amino acid (BCAA) from high‐quality protein are the key nutrients needed to promote muscle growth, while *P*. *cocos* extract has the ability to promote nutrient absorption. Hence, the combination of *P*. *cocos* extract and protein powder may be able to maximize muscle increases during weight loss, bringing longer term health benefits to the body. This study aimed to investigate the effects of *P*. *cocos* and a high‐protein mixture on weight loss and glucolipid metabolism in high‐fat diet‐induced obese mice and their potential mechanisms, and to provide a reliable scientific basis for the wide application of these ingredients.

## 2 MATERIALS AND METHODS

### 2.1 Source of materials

The Beijing *Yili* Technology Development Co., Ltd. provided the interventions used for gavage feeding. The *P*. *cocos* extract came from the patented raw material Lipucan®of XINPHAR GROUP. The protein powder mixture was mainly composed of casein, whey protein, and soy protein isolate in the proportion of 2:1:1.The former two were derived from dairy products, the latter are isolated from soybeans, and the purity of each was over 90%.

### 2.2 Ethical approval statement

All experimental protocols adhered to the guidelines of the Institutional Animal Care and Use Committee of the Laboratory Animal Center of Xiamen University and the International Association of Veterinary Editors guidelines for the Care and Use of Laboratory Animals. Before conducting the experiments, the animals were reviewed and approved by the Animal Ethics and Welfare Committee of the Xiamen University Laboratory Animal Center (approval number: XMULAC20200185).

### 2.3 Animal experiments

C57BL/6J male mice (*n* = 192), mean weight of 19.02 ± 0.88 g, were provided by Shanghai Slack Laboratory Animal Company, Limited. Animals were maintained at a temperature of 22°C and humidity of 10%–60%, 12 h/12 h light–dark cycle, with free access to water. The number of animals was determined based on the success rate of modeling in our previous study (Hsieh et al., 2021), and the sample size was calculated according to Mera et al. (1998). After 1 week of acclimatization, the random number method was used to divide the mice into a blank control group (ND group, *n* = 12) and an obesity model group (*n* = 180), which were fed normal chow (Beijing Keao Xieli Feed Co., Ltd.; Beijing Feed Certificate (2018) 0673) and a high‐fat diet (Research Diets, Inc., D12492), respectively, for 8 weeks. The high‐fat diet [Su Feed Certificate (2017) 05005], which provided 60% of the total calories from fat, was provided by the Jiangsu Shuangshi Laboratory Animal Feed Department. After 8 weeks, mice that met the modeling requirements were further randomized into a model control group (HFD group) and five intervention groups (FL, PM, F1PM, F2PM, and F3PM). Mice with a 40% increase in body weight were considered eligible for inclusion in the study.

During the 10‐week intervention period, the gavage doses for the individual *P*. *cocos* extract and the protein powder alone groups were calculated at 10 times the recommended human dose, which is an approximate range based on human and animal dose conversion, a standard based on human and animal body surface area. The other three combined intervention groups varied based on the *P*. *cocos* extract dose (Table 1). The ND and HFD groups were administered the same volume of saline by gavage, with all mice being gavaged at 0.15 ml/10 g body weight.

**TABLE 1:** Intervention ingredients and formulas.

Group	Test substance	Recommended dose for humans [mg/d]	Intervention dose in mice [mg/d.kg]	Gavage concentration [mg/ml]
ND	Normal saline	—	—	—
HFD	Normal saline	—	—	—
FL	*Poria cocos* extract	66.67	666.67	44.45
PM	Protein powders	333.33	3333.33	222.23
F1PM	*P*. *cocos* extract	16.67	166.67	11.12 + 222.23
	Protein powders	333.33	3333.33	
F2PM	*P*. *cocos* extract	33.33	333.33	22.23 + 222.23
	Protein powders	333.33	3333.33	
F3PM	*P*. *cocos* extract	66.67	666.67	44.45 + 222.23
	Protein powders	333.33	3333.33	

The body weight and food and energy intake of the mice were recorded once per week, 11 times in total. Additionally, the fasting blood glucose (FBG) levels were measured at weeks 0, 4, 6, and 10. At the end of the intervention (week 10), mice (*n* = 8) in each group were randomly selected for oral glucose tolerance tests (OGTT). At the beginning of the OGTT, mice fasted for 12 h were gavaged with 20% glucose solution (10 μl/g) based on body weight, and blood glucose concentrations were determined using tail vein blood at 0, 30, 60, and 120 min after dosing. Sanuo Biological Sensing Co. Ltd provided blood glucose meters utilized in this study.

### 2.4 Body composition analysis

Eight mice randomly selected in each group after intervention were used to determine total adiposity and lean tissue mass using an EchoMRI‐100 h body composition analyzer (Huijia Biological Co., Ltd.).

### 2.5 Monitoring of animal metabolism

Four mice, randomly selected after the intervention, were used to measure respiratory entropy metabolism and voluntary movements using the TES PhenoMaster (12‐channel) system. During the four‐day monitoring period, each mouse was housed singly and fed ad libitum (ND group: standard chow; others: high‐fat chow). The first two of the 4 days were adaptation periods during which data were not collected. Full‐fledged data were collected from 10:00 am on the third day, with data recorded every 5 min, and collection stopped after 48 h.

The respiratory exchange rate (RER) and energy expenditure (EE1) of mice were derived from the consumption of oxygen (O_2_) and the excretion of carbon dioxide (CO_2_) in each confined rearing cage. Furthermore, EE2 was calculated to counteract the impact of weight on energy metabolism, and EE3 was calculated to counteract the impact of weight and lean tissue.

### 2.6 Tissue sampling and index testing

Mice were gas‐anesthetized with 4% isoflurane before execution and sampling. This was followed by eyeball removal for blood collection and execution by rapid cervical dislocation. The mice were dissected, stripped of visceral fat and liver tissue, washed off the blood, and weighed again. The blood was centrifuged at 2000 rpm (382 g) at 4°C for 15 min, and the upper plasma layer was collected for biochemical measurements. All samples were stored frozen at −80°C before being used.

#### 2.6.1 Lipid body ratio

See Li et al. (2019).

$$\text{Lipid body ratio}\left(\%\right)=\left[\text{Peritesticular}\;\mathrm{fat}\;\text{mass}\left(g\right)+\text{Perirenal}\;\mathrm{fat}\;\text{mass}\left(g\right)\right]÷\text{Body mass}\left(g\right)\times 100\%$$

#### 2.6.2 Blood lipids

Serum was collected using an automatic biochemical analyzer (Minray BS‐220) and supporting kits to detect total cholesterol (TC), triglyceride (TG), very low‐density lipoproteins (VLDL), and high‐density lipoproteins (HDL).

#### 2.6.3 Serum insulin (INS), leptin (LEP), adiponectin (ADP), interleukin‐1β (IL‐1β), and tumor necrosis factor alpha (TNF‐α)

Serum INS, LEP, ADP, IL‐1β, and TNF‐α levels were determined using a mouse enzyme‐linked immunosorbent assay kit (ELISA, Shanghai Sanyan Biotechnology Center). Additionally, the insulin resistance index (HOMA‐IR) and islet β‐cell function (HOMA‐β) were calculated using previously reported equations (Matthews et al., 1985; Toin et al., 2022).

#### 2.6.4 Glucose and lipid metabolism‐related factors in the liver

The livers were homogenized in saline and centrifuged at 3000 rpm for 10 min. Supernatants were used to detect the protein expression of lipoprotein lipase (LPL), fatty acid synthase (FAS), sterol regulatory element‐binding transcription factor (SREBP‐1c), and cholesterol 7α‐hydroxylase (CYP7A1) in the liver tissue using an ELISA kit (Shanghai Sanyan Biotechnology Center).

### 2.7 Hematoxylin and eosin (HE) staining of paraffin sections

The morphological changes of liver adipose tissue were observed under a microscope, after the fresh tissues of mice were fixed with 4% formaldehyde, dehydrated, embedded in paraffin, 4 μm thick sections obtained, and HE stained.

### 2.8 Statistical analysis

Data were analyzed using SPSS version 26.0 (IBM Corporation). All results are expressed as the mean ± standard error or median (percentiles). Repeated‐measures univariate analysis of variance (ANOVA) was used to assess the body weight, FBG, and OGTT alterations in mice. If the data distribution was normal and the variance was homogeneous, a one‐way ANOVA was used to compare the data between groups of one‐way measurement indicators, and the LSD method was used for pairwise comparisons between groups. If the data distribution did not satisfy the normal distribution, the nonparametric Kruskal–Wallis H test was used for comparison between groups, and the Nemenyi test was used for pairwise comparison of the overall mean between groups. Statistical significance was set at a 95% probability decision boundary (***p*** < .05).

## 3 RESULTS

### 3.1 Feed intake and body weight

**FIGURE 1:**
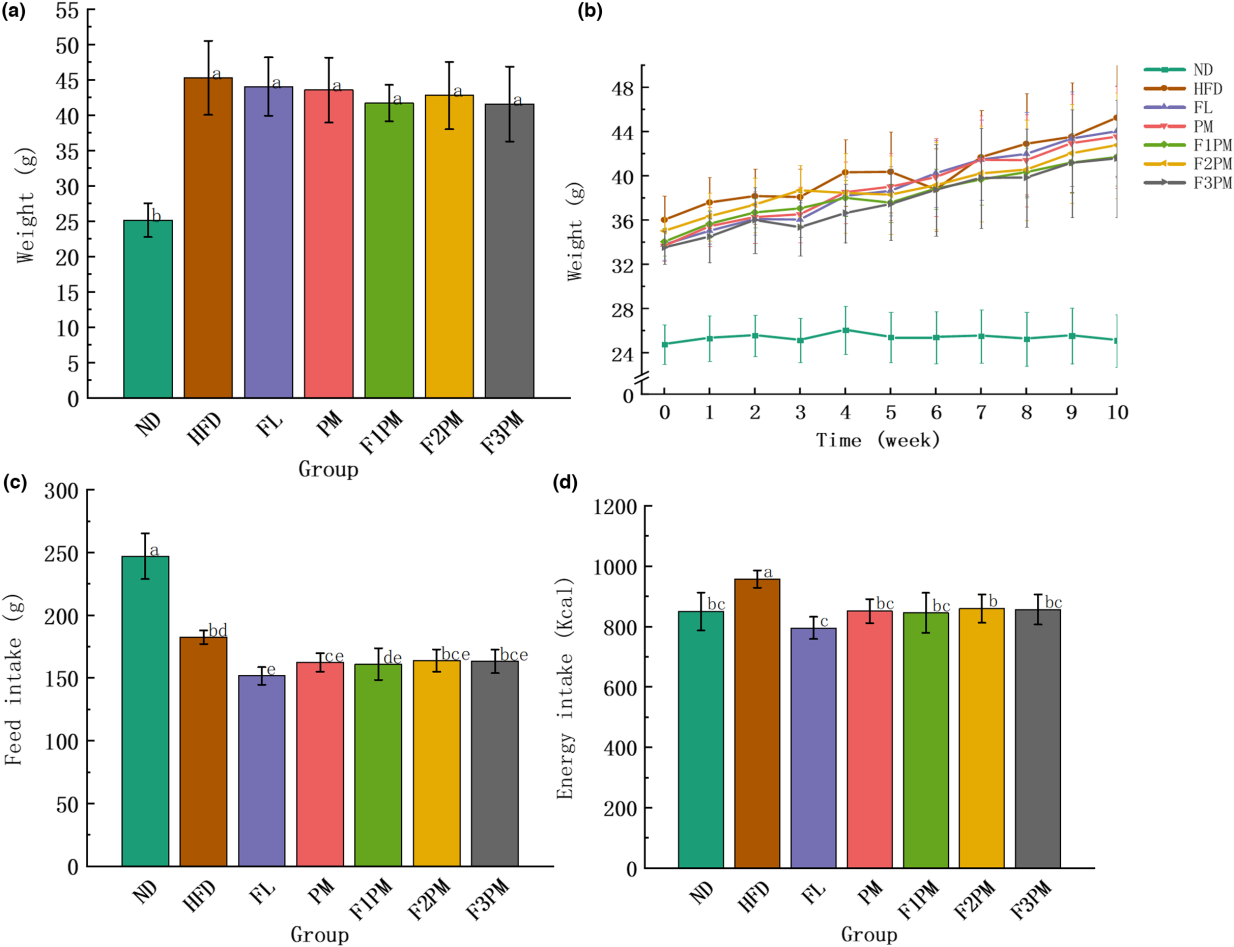
Results of body weight and feed intake. (a) End‐of‐intervention body weight in each group; (b) Body weight during intervention; (c) Total feed intake; (d) Total energy intake; Data are means ± SD (*n* = 12). Different lower case letters above columns indicate statistical differences at *p* < .05.

The mice were re‐grouped in the 0th week before intervention. ND and HFD mice had average body weights of 34.35 ± 1.49 g and 24.79 ± 1.76 g, respectively. The difference was significant, indicating that the high‐fat diet‐induced obesity mouse model had been established successfully. The obese rats were randomly divided into groups, and no significant difference in body weight was seen among the groups, indicating that the grouping into intervention groups was reasonable. The average total food intake per mouse was lower in each intervention group than in the HFD group; however, there was no significant difference among the intervention groups (p > .05). Each mouse in the intervention group had a significantly lower average total energy intake than the HFD group (*p* < .05, Figure 1), especially in the FL intervention group. During the intervention, the body weight of the ND group was consistently lower than that of the intervention and HFD groups. In addition, the weights of the FL, F1PM, and F3PM groups were significantly lower than that of the HFD group during the first 5 weeks of the intervention. Six weeks after the end of the intervention, the weight change trend in each subject group was similar to that of the HFD group, and the weight gain in each intervention group was slightly lower than that of the HFD group (*p* > .05). The body weight of the *P. cocos* extract and protein powder combined intervention group was slightly lower than that of the single intervention groups (*p* > .05). Moreover, no dose–effect relationship was observed in the combined intervention group. In summary, the *P. cocos* alone and *P. cocos* combined intervention could effectively control the weight gain of high‐fat diet‐induced obese mice in the early intervention stage, and there was a long‐term control and maintenance. However, there was little difference in overall weight loss among the groups, especially in the later intervention stage.

### 3.2 Peri‐testicular and peri‐renal fat weights

**FIGURE 2:**
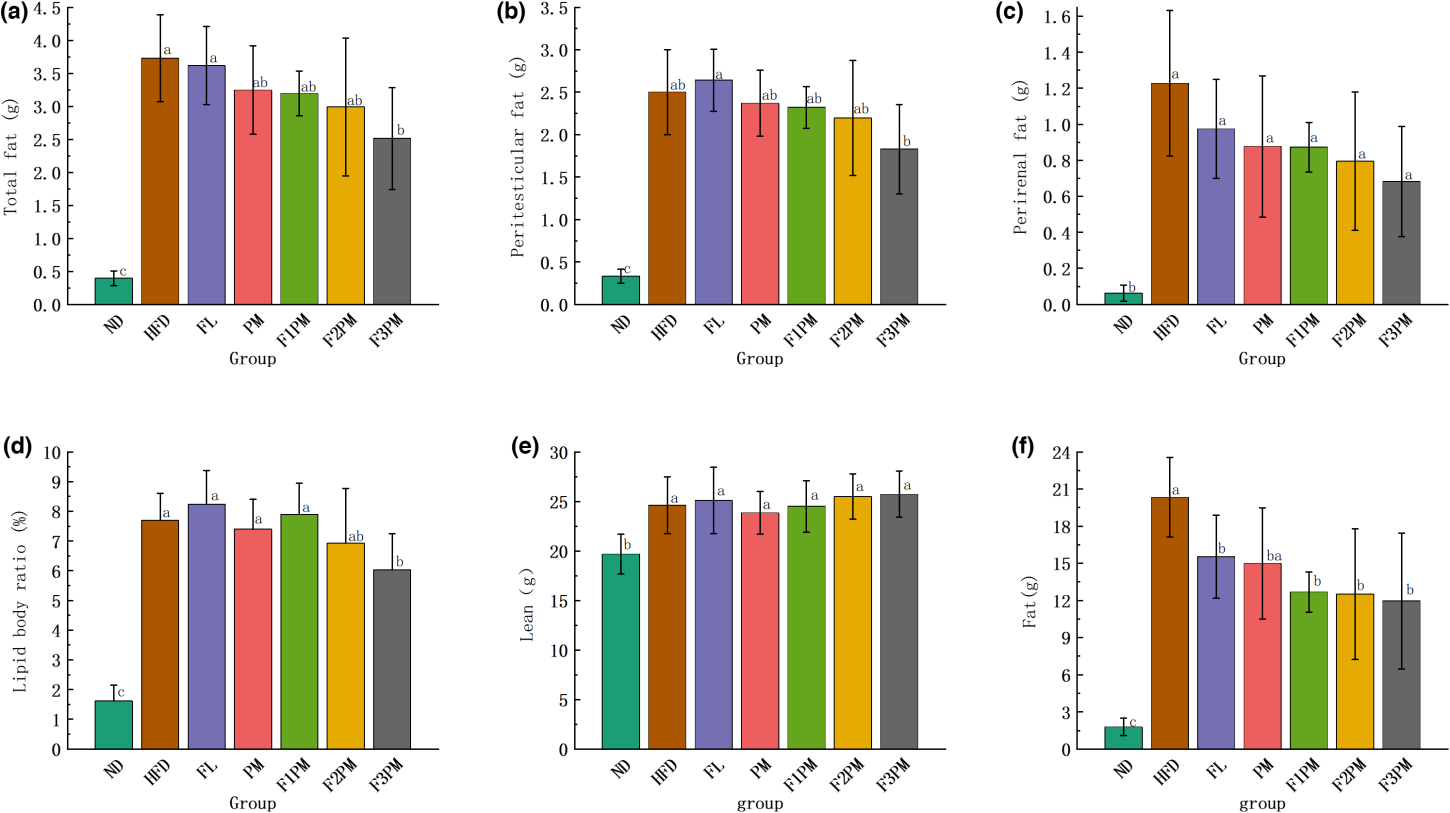
Results of the distribution of body fat and lean tissue. (a) Total fat in each group; (b) Peritesticular fat; (c) Perirenal fat; (d) Lipid body ratio; (e) Lean weight; (f) Fat weight. Data are means ± SD (*n* = 12). Different lower case letters above columns indicate statistical differences at *p* < .05.

Peri‐testicular and peri‐renal fats were 7.51 and 19.58 times higher in the HFD group than in the ND group, respectively, indicating that a high‐fat diet increased animal fat. After the intervention with the test substance, total and peri‐renal fats in each intervention group were lower than in the HFD group, and total fat in the F3PM group decreased significantly (*p* < .05, Figure 2). The total fat content of F3PM group decreased by about 1.22 g, vs. 3.73 g in the HFD mice group. Except for the FL group, the peri‐testicular fat of mice in each intervention group was lower than that in the HFD group after the intervention (*p* < .05). The lipid to body weight ratio of mice in each group was similar to that of the peri‐testicular fat. Fat showed a decreasing trend with an increase in the proportion of *P. cocos* in the combined intervention with *P. cocos* extract and protein powder. However, the difference was insignificant and did not indicate a dose–effect relationship.

### 3.3 Lean and fat contents in the body

A comprehensive assessment of the body composition distribution based on lean and fat tissue content in mice was conducted. After the intervention, the HFD group and all intervention groups had higher lean tissue content than the ND group (*p* < .05, Figure 2). Moreover, the FL, F2PM, and F3PM groups had slightly higher lean tissue content than the HFD group. The combined intervention group demonstrated an increasing trend in lean tissue, but the difference was insignificant. The HFD group had significantly increased body fat content (*p* < .05). After 10 weeks of intervention, the fat content was significantly lower in all intervention groups but significantly higher than the mean of the ND group (*p* < .05).

### 3.4 Liver weight and morphological structure of the liver tissue

**FIGURE 3:**
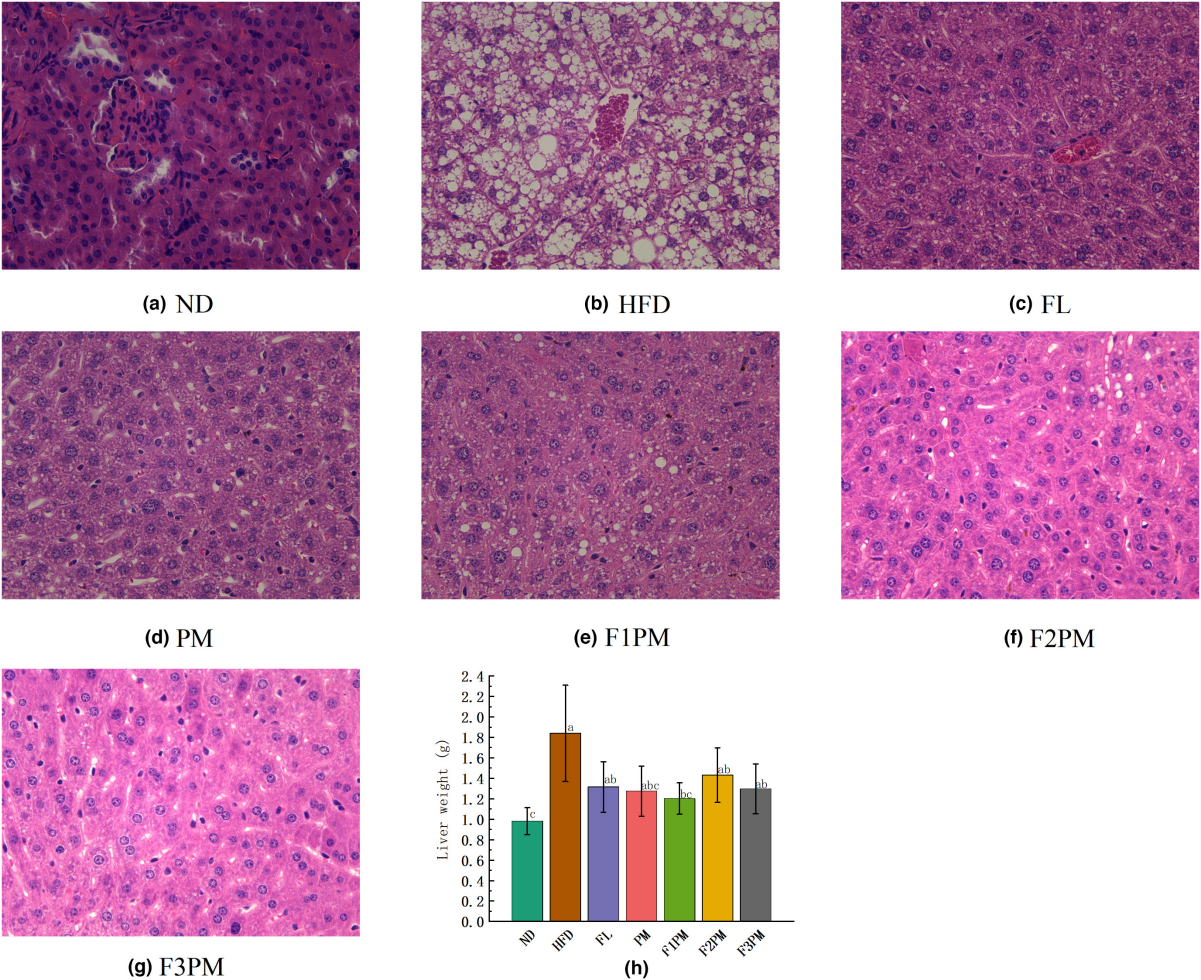
Liver weight and morphological structure of the liver tissue. (a) HE staining of liver tissue in ND group; (b) HE staining of liver tissue in HFD group; (c) HE staining of liver tissue in FL group; (d) HE staining of liver tissue in PM group; (e) HE staining of liver tissue in F1PM group; (f) HE staining of liver tissue in F2PM group. (g) HE staining of liver tissue in F3PM group; (h) Liver weight. Data are means ± SD (*n* = 12). Different lower case letters above columns indicate statistical differences at *p* < .05.

The liver structure of the ND group was normal, with the hepatic plate and sinusoid arranged radially and orderly (Figure 3). The HFD group*'*s liver weight was significantly higher than the ND group's (*p* < .05). The liver structure was disordered, and numerous lipid droplet vacuoles of different sizes appeared in the cytoplasm, resulting in steatosis. After the intervention with the test substance, the liver weight of each group decreased, the number of lipid droplets decreased greatly, and the liver structure was effectively improved, especially in the F2PM and F3PM intervention groups.

### 3.5 Blood lipid level

**FIGURE 4:**
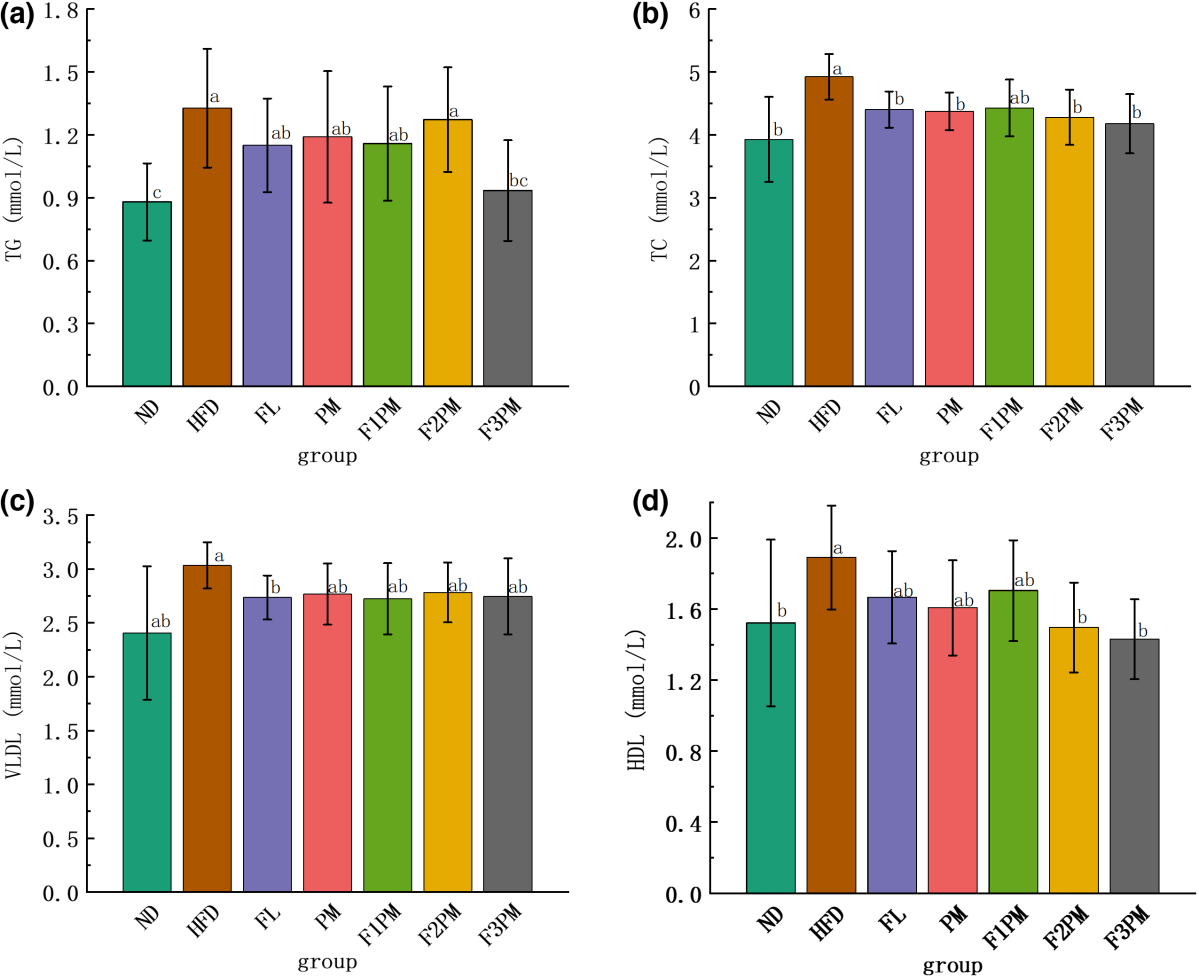
Blood lipid metabolism index. (a) TG (triglycerides); (b) TC (total cholesterol); (c) VLDL (very low‐density lipoprotein); (d) HDL (high‐density lipoprotein); Data are means ± SD (*n* = 12). Groups that do not share the same letter are significantly (*p* < .05) different from each other.

Serum levels of TG, TC, VLDL, and HDL increased in mice after high‐fat modeling. After the intervention with the test substance, the four blood lipid items in each intervention group were lower than in the HFD group. In each subject group, the TC, VLDL, and HLDL levels were similar to those in the ND group (*p* > .05, Figure 4), whereas the TG indicators remained higher in the intervention groups than in the ND group, except for F3PM. Regardless of the difference in *P*. *cocos* extract and protein powder mixture dose, no significant difference in blood lipid levels between the combined intervention groups was observed (*p* > .05).

### 3.6 FBG level

**FIGURE 5:**
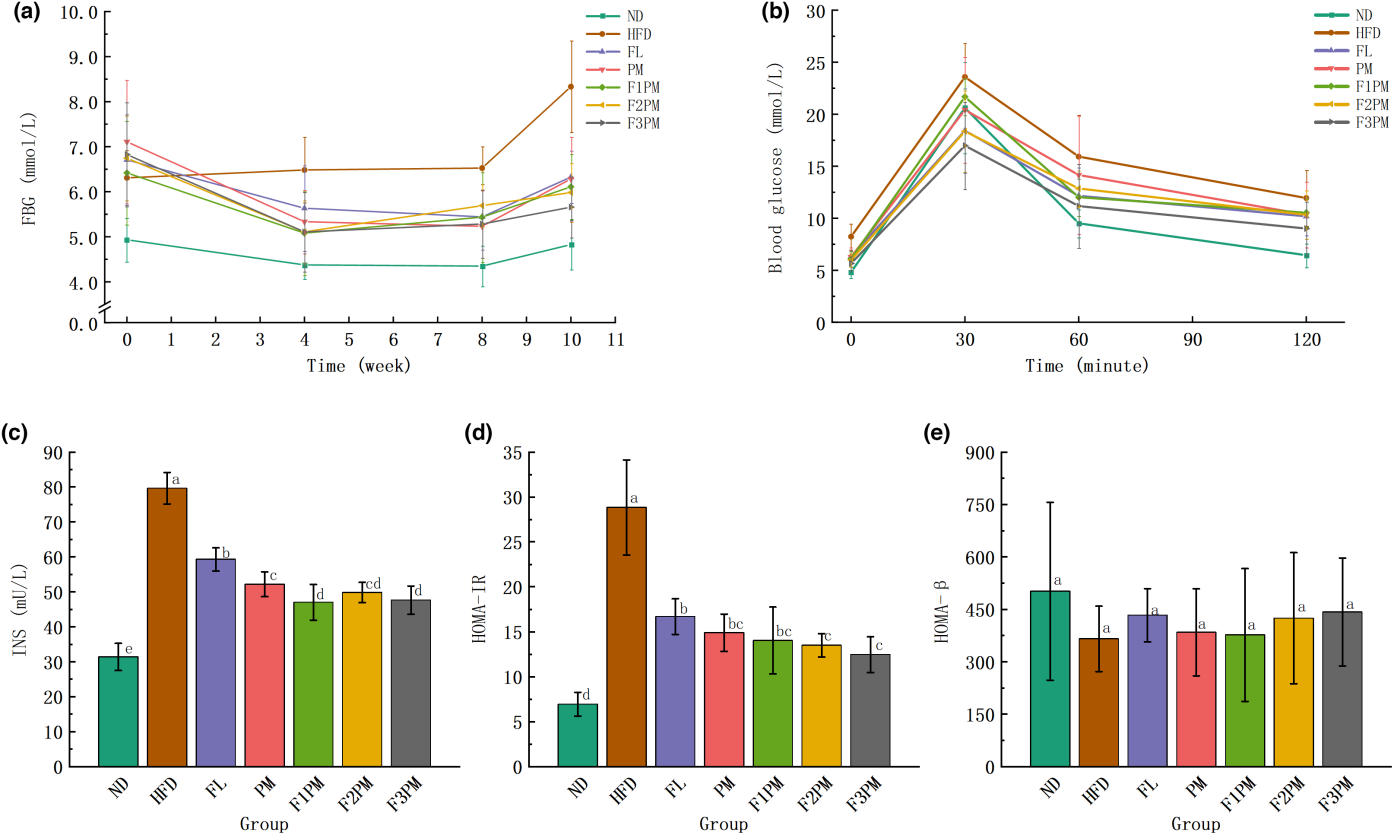
Results of markers of glucose metabolism. (a) Fasting blood glucose; (b) OGTT (Oral glucose tolerance tests); (c) Serum INS (insulin) level. (d) HOMA‐IR (insulin resistance index); (e) HOMA‐β(insulin β‐cell function); Data are means ± SD (*n* = 12); Groups that do not share the same letter are significantly (*p* < .05) different from each other.

During the intervention period, the fluctuation of blood glucose in the ND group was small and maintained at the same level. The blood glucose level in the model control group increased slowly, and the FBG level in each intervention group decreased. This indicates that all interventions effectively controlled the elevated FBG levels caused by high‐fat diets. At the 0th week of intervention, blood glucose levels were greater than 6.1 mmol/L in all intervention and HFD groups, with no difference between groups, and FBG was higher than in the ND group (*p* < .05, Figure 5). From the 4th week to the end of the intervention, the FBG level of each intervention group was lower than that of the HFD group, and the difference was significant. At different time points, the blood glucose levels in each intervention group were the same, and the difference was insignificant. This shows that using *P*. *cocos* extract and protein powder mixture, either alone or in combination, can quickly adjust FBG to a relatively normal level while providing a long‐term and stable glucose control effect.

### 3.7 OGTT test

The area under the OGTT curve (AUC) of each intervention group was smaller than that of the HFD group, indicating that all interventions enhanced glycemic regulation in obese mice (Figure 5). When treated with *P*. *cocos* extract and protein powder alone, the relative AUC of the FL group was lower than that of the PM group. However, in the combined intervention group, the relative AUC value decreased as the proportion of *P*. *cocos* increased. This suggests that *P*. *cocos* extract was more effective than protein powder mixtures in controlling blood sugar levels.

### 3.8 Serum INS, HOMA‐IR, and HOMA‐β levels

INS and HOMA‐IR levels were elevated in the HFD group compared with the ND group (*p* < .05, Figure 5), suggesting that the high‐fat diet‐induced obese rats compensated for insulin secretion and possibly insulin resistance due to elevated glucose. After the intervention, INS and HOMA‐IR levels decreased significantly in all groups (*p* < .05). HOMA‐β levels increased after the intervention compared with the HFD group (*p* > .05). All interventions, either alone or in combination, attenuated HFD‐induced islet damage. Any differences in the effect between the combined intervention groups with different doses of *P*. *cocos* extract and protein powder were insignificant.

### 3.9 Indicators related to energy metabolism

**FIGURE 6:**
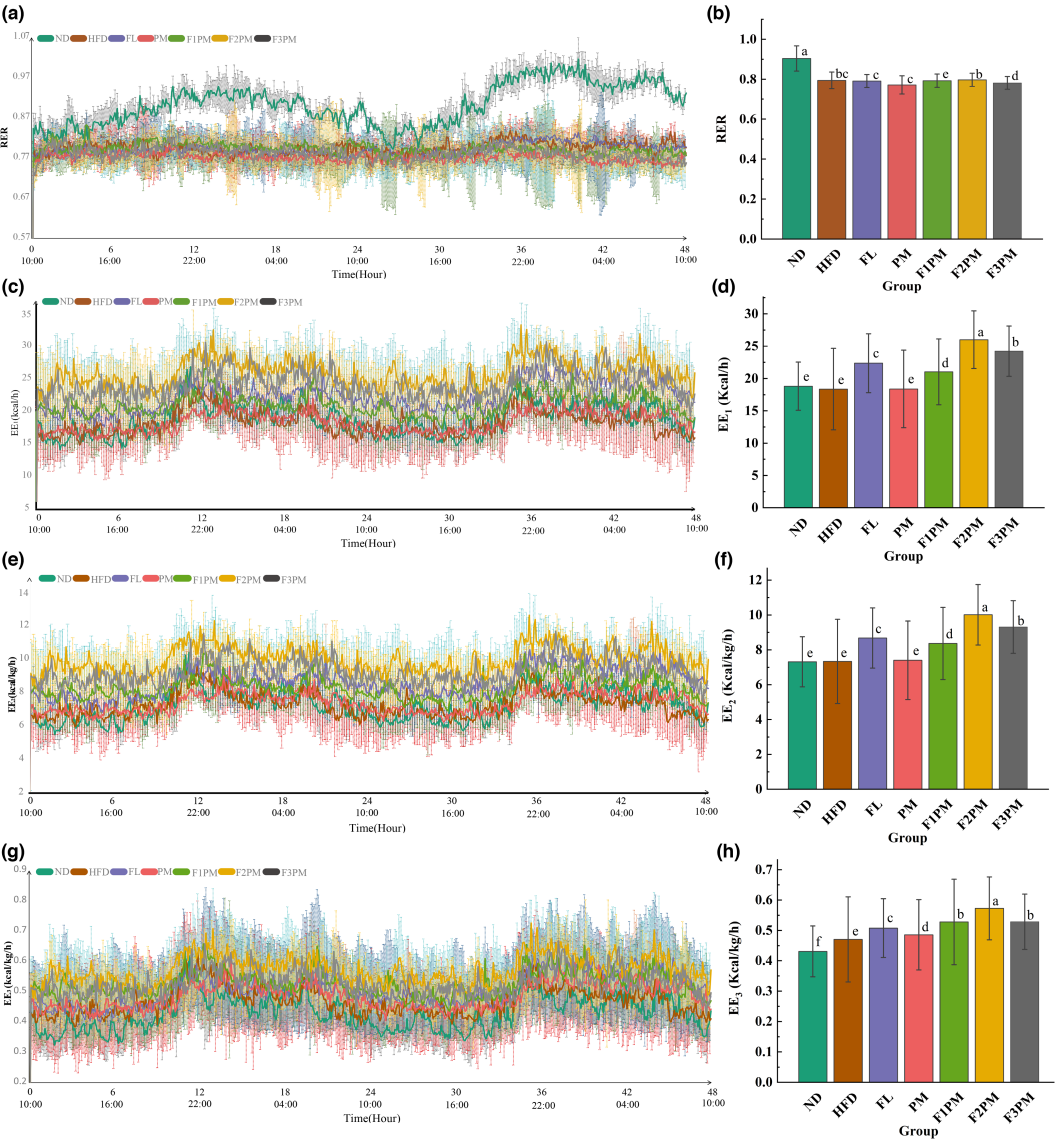
Energy metabolism‐related indicators. (a) Change in the respiratory exchange ratio (RER) over time; (b) Comparison of the mean RER of each group; (c) Change in the energy expenditure (EE1) over time; (d) Comparison of the mean EE1 of each group; (e) Change in the energy expenditure (EE2) over time; (f) Comparison of the mean EE2 of each group; (g) Change in the energy expenditure (EE3) over time; (h) Comparison of the mean EE3 of each group; The body weight was used to correct EE1 to obtain EE2, and the lean body mass was used to correct EE2 to obtain EE3. Data are means ± SD (*n* = 12); Groups that do not share the same letter are significantly (*p* < .05) different from each other.

The VCO_2_/VO_2_ ratio, also known as RER, is often used to assess energy‐supplying substance use. As shown in Figure 6, the RER of mice in the ND group exhibited a circadian rhythm by fluctuating between 0.80 and 1.0 and being higher at night than during the day. The RER of mice in the HFD and subject intervention groups showed no circadian rhythm and was maintained at approximately 0.80. The ND group had the highest RER (*p* < .05), the PM group had the lowest RER (*p* < .05), the FL and F1PM groups had similar RER to the HFD group (*p* < .05), and the F2PM group had a higher RER than the HFD group (*p* < .05). This indicates that obesity in mice caused by a high‐fat diet can change the magnitude and rhythm of respiratory entropy, and that subject intervention can change the RER level in obese mice; protein powder alone can reduce the RER, while the combined intervention of protein powder and *P*. *cocos* extract can increase the RER.

**FIGURE 7:**
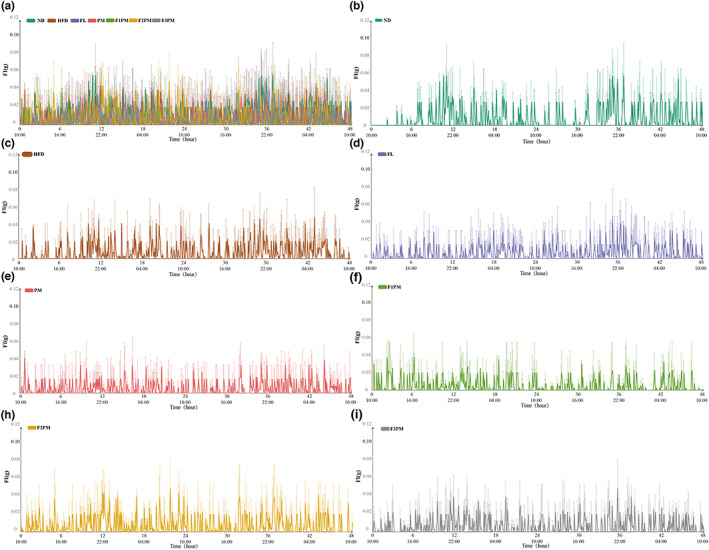
Changes in food intake over time; (a) Changes of food intake in each group; (b) Changes of food intake in ND group; (c) Changes of food intake in HFD group; (d) Changes of food intake in FL group; (e) Changes of food intake in PM group; (f) Changes of food intake in F1PM group; (h) Changes of food intake in F2PM group; (i) Changes of food intake in F3PM group.

As shown in Table 2, the intake of each intervention group was insignificant to that of the HFD group. However, as shown in Figure 7 of intake–time variation, the intake of mice in the ND group was consistent with the variation of RER with a circadian rhythm that was higher at night than during the day. No feeding rhythms were observed in the HFD group. After the intervention, it was discovered that the feeding rhythm recovered in the FL group but not in the PM group. With an increase in the dose of *P*. *cocos* extract, the feeding rhythms of F1PM, F2PM, and F3PM were closer to that of the ND group. This indicates that normal mice have a feeding rhythm, and obesity changes the feeding habits of mice, disrupting the rhythm of feeding. *P*. *cocos* intervention can restore the rhythm of feeding in obese mice. While protein powder intervention alone has not been shown to restore the feeding rhythm, the combination of intervention with *P*. *cocos* may accomplish this.

**TABLE 2:** Result of Energy metabolism‐related indicators.

Group	Test substance	RER $\left(\bar{X}\pm S\right)$	EE1 ($\bar{X}\pm S$, kcal/h)	EE2 ($\bar{X}\pm S$, kcal/kg/h)	EE3 ($\bar{X}\pm S$, kcal/kg/h)	FI	Amount of exercise
ND	Normal saline	0.9 ± 0.06^a^	18.8 ± 3.73^e^	7.31 ± 1.44^c^	0.43 ± 0.08^c^	2.22 ± 0.18^a^	1287.71 ± 229.92^a^
HFD	Normal saline	0.79 ± 0.04^bc^	18.36 ± 6.3 ^e^	7.34 ± 2.42^f^	0.47 ± 0.14^f^	2.01 ± 0.27^a^	1379.95 ± 109.12^a^
FL	*Poria cocos* extract	0.79 ± 0.03^c^	22.37 ± 4.55^c^	8.68 ± 1.73 ^e^	0.51 ± 0.1^g^	1.75 ± 0.37^a^	1372.5 ± 295.29^a^
PM	Protein powders	0.77 ± 0.05^e^	18.38 ± 5.99^e^	7.4 ± 2.25^f^	0.49 ± 0.12^d^	1.48 ± 0.85^a^	1199.85 ± 141.87^a^
F1PM	*P*. *cocos* extract	0.79 ± 0.03^c^	21.03 ± 5.1^d^	8.37 ± 2.07^d^	0.53 ± 0.14^e^	1.4 ± 0.41^a^	1123.46 ± 462^a^
	Protein powders						
F2PM	*P*. *cocos* extract	0.8 ± 0.03^b^	26 ± 4.45^a^	10.02 ± 1.7^b^	0.57 ± 0.1^a^	2.16 ± 0.16^a^	1108.42 ± 174.71^a^
	Protein powders						
F3PM	*P*. *cocos* extract	0.78 ± 0.03^d^	24.23 ± 3.89^b^	9.31 ± 1.51^a^	0.53 ± 0.09^b^	1.84 ± 0.43^a^	895.98 ± 261.92^a^
	Protein powders						

*Note*: Groups that do not share the same letter are significantly (*p* < 0.05) different from each other.

As shown in Figure 6, energy metabolism in mice has a circadian rhythm, and both normal and obese mice consume more energy at night than during the day. Energy expenditure values differed among the groups, with or without body weight and lean tissue regulation. The FL, F1PM, F2PM, and F3PM groups had higher energy expenditure than the HFD group (*p* < 0.05), with the F2PM group having the highest (*p* < 0.05), and the PM group being the most similar to the HFD group (*p* > 0.05). Combined with the results of food intake and exercise, it can be speculated that *P*. *cocos* alone or in combination with protein may improve energy expenditure by increasing basal metabolic values in mice, and that combination is more efficacious than *P*. *cocos* extract or protein powder alone.

### 3.10 Serum inflammatory factors, IL‐1β and TNF‐a

**FIGURE 8:**
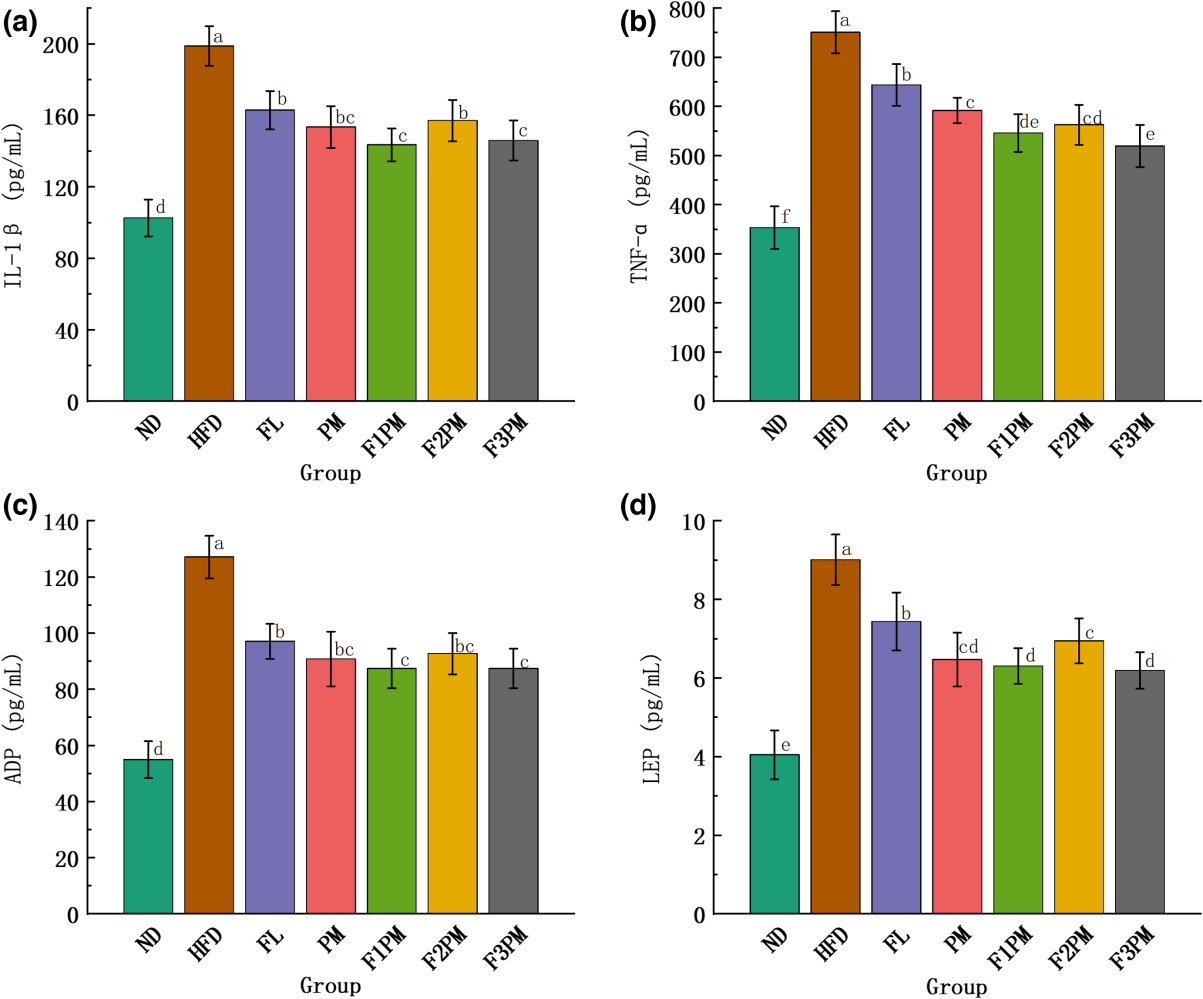
Results of serum glycolipid metabolism‐related indicators; (a) Inflammatory factors IL‐1β; (b) Inflammatory factors TNF‐α; (c) Serum ADP (Adiponectin) level; (d) Serum LEP (Leptin) level; Data are means ± SD (*n* = 12); Groups that do not share the same letter are significantly (*p* < .05) different from each other.

As shown in Figure 8, serum IL‐1β levels were higher in the HFD group than in the ND group, whereas serum IL‐1β levels were lower in all intervention groups than in the HFD group (*p* < .05). The results of this study suggest that high‐fat diets increase serum IL‐1β levels in obese mice, and that subject intervention can downregulate serum IL‐1β levels and improve the chronic inflammatory state caused by high‐fat diets. The change in serum TNF‐α level was similar to that of IL‐1β. This study demonstrated that each intervention could effectively downregulate TNF‐α level, and that the combined intervention was more effective than *P*. *cocos* and protein powder alone.

### 3.11 Serum LEP and ADP levels

LEP is a protein‐like hormone secreted by white adipose tissue that regulates feeding behavior, inhibits lipid synthesis, and promotes lipolysis (Münzberg & Morrison, 2015). When LEP levels rise, LEP receptors decrease, leading to LEP resistance, a risk factor for obesity (Myers et al., 2008). As shown in Figure 8, the serum LEP levels among the different groups differed significantly. Serum LEP levels in the HFD group were higher than those in the ND group, and serum LEP levels in each subject group were lower than those in the HFD group, indicating that serum LEP levels increased in mice with obesity caused by high‐fat diets and that subject intervention could downregulate serum LEP levels.

ADP is a peptide or protein secreted by adipocytes that plays an important messenger role in the communication between adipose tissue and other organs. As shown in Figure 8, serum ADP levels in each intervention group were lower than that in the HFD group (*p* < .05), indicating that the intervention may downregulate serum ADP levels and effectively improve fat metabolism. In addition, regardless of LEP or ADP level, there was no significant difference between the groups in the combined intervention group and no significant advantage compared with the single intervention group.

### 3.12 LPL, CYP7A1, FAS, and SREBP‐1c levels in liver tissue

**FIGURE 9:**
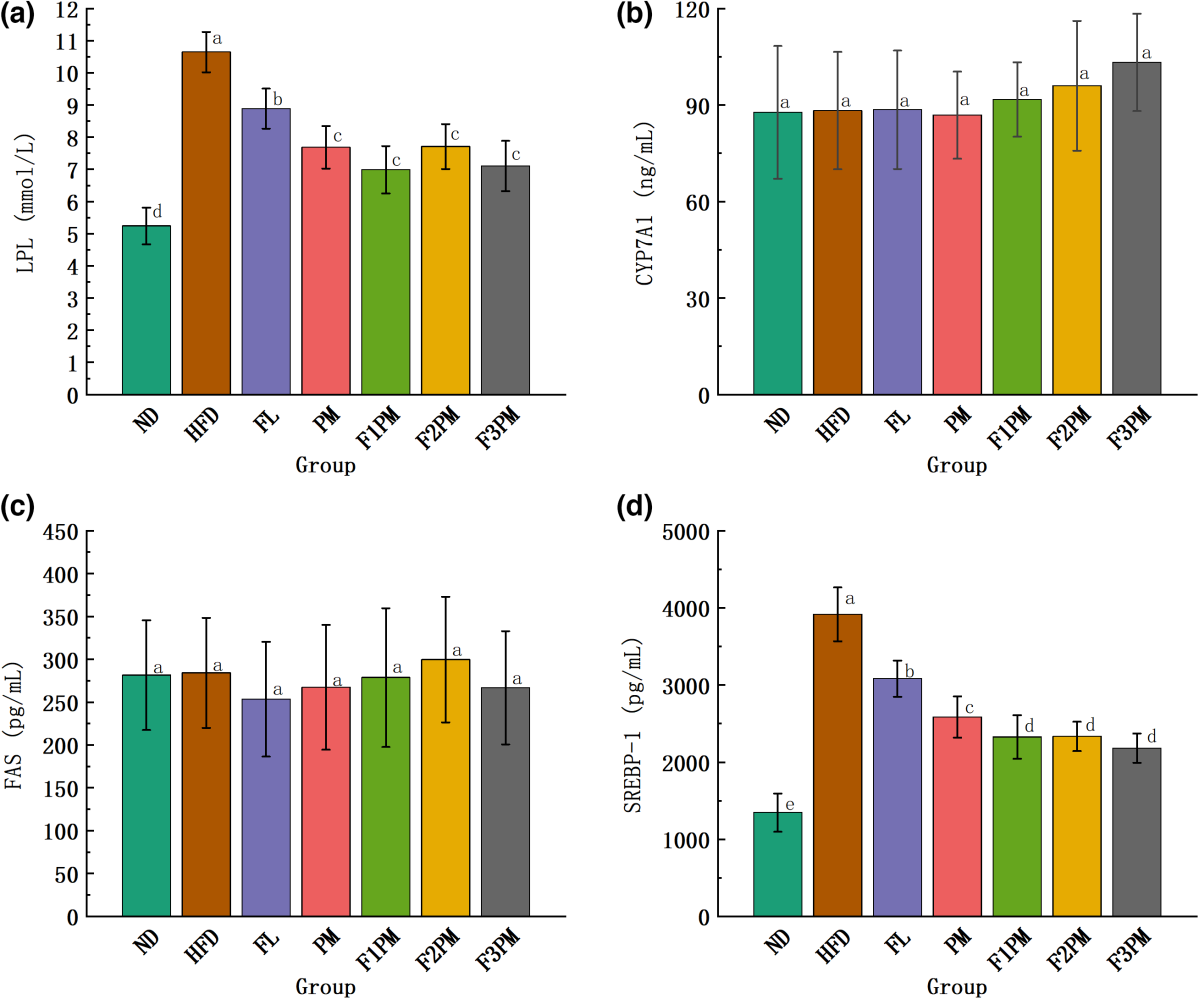
Results of glycolipid metabolism‐related indicators in liver tissue. (a) LPL (lipoprotein lipase); (b) CYP7A1 (cholesterol7α‐hydroxylase); (c) FAS (fatty acid synthase); (d) SREBP‐1 (Sterol regulatory element‐binding transcription factor‐1); Data are means ± SD (*n* = 12); Groups that do not share the same letter are significantly (*p* < .05) different from each other.

LPL, CYP7A1, FAS, and SREBP‐1c are key enzymes in lipid synthesis and metabolism in vivo. As shown in Figure 9, LPL and SREBP‐1c levels in the liver tissues of mice in each intervention group were lower than those of HFD mice (*p* < .05), indicating that the high‐fat diet caused overexpression of LPL and SREBP‐1c in the liver tissues of obese mice. In contrast, the intervention reduced the levels of LPL and SREBP‐1c. There were no significant differences in CYP7A1 and FAS levels among the groups, indicating that the intervention of the tested substance did not affect CYP7A1 and FAS expression.

## 4 DISCUSSION

Obesity is an endocrine, metabolic disease characterized by excessive accumulation of body fat and abnormal fat distribution. Its pathogenesis involves various factors, such as diet, genetics, metabolism, and hormone secretion, which interact with each other to cause an energy imbalance in the body. Most of the excess energy ingested by the body is stored as fat, or adipose tissue. However, excessive lipid accumulation can dysregulate the microenvironment of fat depots and affect peripheral tissue metabolism (Booth et al., 2015). Adipose tissue is a complex organ that can reserve energy and has endocrine, metabolic, and immunomodulatory effects. Furthermore, the secretion of various hormones and inflammatory products into the body can disrupt glucolipid metabolism and trigger chronic stress reactions. In this study, mice in the HFD group had significant fat accumulation and metabolic disorders due to the long‐term intake of a high‐fat diet. After 10 weeks of *P*. *cocos* and protein powder intervention, there was no significant difference in the body weight of mice in each test subject group, while voluntary food intake and total energy intake were significantly lower compared with the HFD group. This suggests that the *P*. *cocos* extract and protein powder intervention may, to some extent, increase satiety and reduce dietary intake. In addition to the specific body composition analysis, it is evident that the fat weight in the subject group decreased significantly during the intervention period, with more significant changes in peri‐testicular and peri‐renal fat and a slight increase in lean tissue weight, which was most obvious in the F3PM group. This suggests that *P*. *cocos* and protein powder may reduce fat and increase muscle mass, while no significant changes were observed in overall weight, due to the balance of fat‐muscle weight conversion. In addition, the liver weight of mice in each intervention group was significantly reduced after intervention with *P*. *cocos* extract and protein powder. The pathological sections revealed obvious pathological changes in the liver tissues of the HFD group. In contrast, the hepatocytes of the mice in the intervention group were arranged uniformly and orderly without the aggregation of lipid droplet vacuoles. Moreover, the morphological structure of the liver was similar to that of the ND group, which was significantly improved compared with the HFD group. This indicates that the test substance can effectively treat severe steatosis in the hepatocytes of obese mice, reduce ectopic fat deposition in the liver, alleviate liver function damage, and regulate abnormal liver lipid metabolism. From this perspective, the combined intervention of *P*. *cocos* and protein powder has advantages over the ketogenic diet, which is a high‐fat diet with limited carbohydrate content. The ketogenic diet is popular because it creates a metabolic shift, but it is also controversial because a high‐fat diet is related to negative health outcomes. Studies have shown that even a short‐term ketogenic diet can induce more severe hepatic insulin resistance than high‐fat diet (Grandl et al., 2018), and the increase of hepatic lipid metabolites can induce hepatic steatosis (Jornayvaz et al., 2010). The *P*. *cocos* and protein powder interventions not only improved fat accumulation and distribution and the steatosis of liver. In addition, blood glucose and lipid levels in all intervention groups decreased to varying degrees, with the F3PM group having a more significant effect on blood glucose and lipid improvement. These improvements may be attributed to the intervention's impact in regulating factors related to glucose and lipid metabolism.

Studies have shown that adipokines are involved in many physiological functions, ranging from satiety regulation to glucose homeostasis. In both obesity and obesity‐related metabolic disorders, dysregulation of adipokine expression exists and excessive fat accumulation can trigger harmful metabolic disorder cascades by altering the distribution of adipokines in the body, increasing the risk of hyperglycemia, insulin resistance, and dyslipidemia in obese patients (Booth et al., 2015). The mechanism of obesity associated with adipokines mainly involves the fact that obesity causes adipose tissue hypertrophy, which in turn causes hypoxia. Hypoxia further causes an inflammatory response that inhibits ADP expression, while increasing the secretion of LEP and related inflammatory factors, which is proportional to obesity, glucose tolerance, and the degree of insulin resistance (Shi & Cheng, 2006). LEP and ADP are hormones produced by adipose tissues that regulate energy intake and expenditure, as well as lipid and carbohydrate metabolism (Havel, 2004). In this study, the serum LEP level increased in high‐fat diet‐induced obese mice and the intervention of the tested substance downregulated the serum LEP level, suggesting that the tested substance could alleviate LEP resistance caused by obesity. In addition, ADP was significantly downregulated in all *P*. *cocos* and protein powder intervention mice compared with HFD mice. Combining the results of lipid and glucose regulation in the experimental mice in each intervention group, it is hypothesized that the effect of subject intervention may be two‐fold. The adipose tissue content reduction in obese mice causes a decrease in ADP secretion. However, reducing insulin resistance in obese mice with a high‐fat diet leads to the downregulation of ADP receptors and a decrease in ADP levels. In addition, we discovered that LPL and SREBP‐1c were overexpressed in HFD mice, and the LPL and SREBP‐1c levels in the liver tissue decreased with the intervention of the tested substances, especially in the combined intervention group, possibly by reducing chronic inflammation, reducing endoplasmic reticulum stress, further regulating related signaling pathways, and reducing enzyme expression. The expression of these enzymes reduces fat synthesis and accumulation.

The RER is an important index for measuring the contribution ratio of carbohydrates and fatty acids as energy sources. An RER closer to 1 indicates the energy source is mainly carbohydrate, whereas a value closer to 0.7 indicates that the substrate of energy metabolism is mostly fat (Virtue & Vidal‐Puig, 2013). Normal metabolism fluctuates between these two values. Some studies have shown that in nonobese subjects, the main metabolic substrate in fasting state is free fatty acid (FFA), and the RQ is about 0.82. During the postprandial state, the metabolic substrate mainly turns to glucose, and the RQ is about 1.00, indicating that glucose is almost the only source of energy. In contrast, in obese subjects, the RQ on an empty stomach was 0.9, and there was no change at all in the postprandial state, indicating that there was no transfer of energy metabolic substrate (Hara et al., 2022). The change of RER can reflect the metabolism of glucose and lipid, and obesity will make the metabolism inflexible. In this study, the RER of mice in the ND group fluctuated between 0.80 and 1.0 with a circadian rhythm, whereas the RER of mice in the HFD group showed no circadian rhythm and was maintained at approximately 0.80, which is consistent with previous studies (Speakman, 2013). Obesity in mice induced by a high‐fat diet may alter the size and rhythm of respiratory entropy. After the intervention, the RER of obese mice increased, indicating that the proportion of carbohydrates as substrates of energy metabolism increased, which may be related to the intervention's ability to reduce serum inflammatory factors (IL‐1 β and TNF‐ α), alleviate insulin resistance, and improve the body's ability to utilize sugar (Zhou et al., 2021). Fat contributes more to energy metabolism in high‐fat‐induced obese mice. A large amount of FFA is produced when oxidized fat is the primary metabolic pathway, and FFAs are closely related to inflammatory factors. Studies have shown that the inflammatory factor TNF‐α can promote FFA release via the hormone‐sensitive lipase pathway, which can increase TNF‐α secretion by macrophages, leading to a vicious circle in which both secretions promote each other (Bouter et al., 2010). Macrophage infiltration in obese mice leads to high expression of inflammatory factors, such as TNF‐α and IL‐1 β, leading to chronic inflammation development. Chronic inflammation is an important factor in the occurrence and development of chronic metabolic diseases (Xia et al., 2021). Studies have shown that with an increase in TNF‐α and IL‐1 β levels, the TC, LEP, and ADP levels increase, suggesting that the levels of inflammatory factors may affect glucose and lipid metabolisms (Xue et al., 2005). TNF‐α is a pro‐inflammatory cytokine that reduces the response of adipocytes, hepatocytes, and muscle cell responses to insulin (Ramírez Alvarado & Sánchez Roitz, 2012). Additionally, TNF‐α is involved in insulin resistance‐induced apoptosis and atrophy of brown adipocytes, promoting the development of hyperinsulinemia combined with obesity and driving the progression of insulin resistance (S. Wang et al., 2021). Furthermore, IL‐1β can promote the release of inflammatory cytokines by mediating pancreatic β‐cell apoptosis or activating the NF‐κB pathway, thereby inducing insulin resistance and the development of type 2 diabetes (Xue et al., 2005; Zhang & Kaufman, 2008). In this study, the inflammatory factors TNF‐α and IL‐1 β decreased significantly, insulin secretion decreased, insulin resistance symptoms alleviated, and the level of HOMA‐β increased in each intervention group. This study demonstrated a significant decrease in TNF‐α and IL‐1β levels, a decrease in insulin secretion, a reduction in insulin resistance symptoms, and an increase in HOMA‐β levels in each intervention group. This indicates that the *P*. *cocos* and protein powder intervention can modulate relevant inflammatory factors and glucose metabolic pathways, as well as improve high‐fat‐induced islet damage. However, there is a limit to these regulatory abilities, and the relevant indexes in all groups failed to recover to the level of the ND group. Therefore, the modulating ability of F3PM was more pronounced in the combined intervention group than in the *P*. *cocos* and protein powder effects alone.

In addition, we observed certain phenomena regarding intake and energy consumption. First, the ND group showed a circadian rhythm in the variation of feeding, while the feeding rhythm was disturbed in the HFD group. The intervention by the subject restored the feeding rhythm, which was more obvious in the intervention group with a higher proportion of *P*. *cocos*. Second, energy metabolism in mice was rhythmic, and intervention increased basal energy metabolism while maintaining circadian rhythms. All mammals exhibit circadian rhythms in their daily activities, which contribute to the maintenance of peripheral tissue metabolism and energy homeostasis (Froy, 2012). Metabolic mediators secreted by adipose tissue (such as ADP and LEP) as well as key metabolic factors in adipocytes, are regulated by rhythms and exhibit diurnal variations (Ando et al., 2005; Bray & Young, 2007). In addition, many hormones that regulate metabolism, including insulin (La Fleur et al., 1999) and glucagon (Ruiter et al., 2003), and corticosterone (De Boer & Van der Gugten, 1987), also exhibit circadian rhythm fluctuations. Interrupting the rhythm of adipokine‐regulated cellular metabolic processes predisposes the organism to obesity and obesity‐related diseases (Hatori et al., 2012). Obesity exacerbates adipose tissue dysfunction and regulates the secretion of pro‐inflammatory factors, leading to chronic low‐grade inflammation, thus enhancing the systemic metabolic disorders associated with obesity. In this study, the animals in each intervention group exhibited good control of blood glucose and lipids and a corresponding reduction in body fat. This suggests that the intervention may help adjust the circadian rhythm disorder induced by a high‐fat diet, affect the secretion levels of related factors, and improve the state of glucose and lipid metabolism.

## 5 CONCLUSIONS

In summary, *P*. *cocos*, protein powder and their combined intervention effectively improved fat distribution, glycolipid metabolism, energy metabolism, and contributed to the recovery of biological rhythm in obese mice. These effects are reflected in the F3PM combined interventional group.

## AUTHOR CONTRIBUTIONS

Conceptualization, Q.X. and X.J.; methodology, Q.X. and X.J.; software, W.Z.; validation, Y.X., M.Z. and Z.Z.; formal analysis, Q.X. and W.Z.; investigation, Q.X., W.Z., M.Z and Y.X.; resources, J.H.; data curation, H.L., J.D. and Y.L; writing—original draft preparation, Q.X.; writing—review and editing, X.J.; visualization, H.F., J.H. and H.L.; supervision, H.F., J.H. and H.L.; All authors have read and agreed to the published version of the manuscript.

## FUNDING INFORMATION

This research received no external funding.

## CONFLICT OF INTEREST

The authors declare no conflict of interest.

## Data Availability

The data that support the findings of this study are available on request from the corresponding author. The data are not publicly available due to privacy or ethical restrictions.
